# Combination therapy with methylprednisolone and rivaroxaban for mild COVID-19 in Vietnam: laboratory and clinical insights

**DOI:** 10.3205/dgkh000567

**Published:** 2025-07-09

**Authors:** Le Thi Anh Thu, Phong Hoang, Tuan Nguyen, Nguyen Phan Hoang, Ngan La Thi Thanh, Thanh Nguyen Thi Hai, Hiep Nguyen Van, Thanh Pham Tuan, Lan Tran Thi Tuyet, Nuong Nguyen Thi My, Truc Lai Thi Thu, Toan Vi Van, Phuong Nguyen Thi, Minh Nguyen Thien

**Affiliations:** 1 Hoan My Academy, Ho Chi Minh City, Vietnam; 2 Hoan My Thu Duc Hospital, Ho Chi Minh City, Vietnam; 3 Van Phuc 2 Hospital, Binh Duong, Vietnam; 4 University of Medicine and Pharmacy at Ho Chi Minh City, Ho Chi Minh City, Vietnam

**Keywords:** COVID-19, Methylprednisolone, Rivaroxaban, mild disease, combination therapy

## Abstract

**Objective:**

To assess the efficacy and safety of methylprednisolone combined with rivaroxaban versus rivaroxaban monotherapy in mild cases of COVID-19.

**Methods:**

This quasi-experimental study was conducted at three hospitals in Ho Chi Minh City, Vietnam, from September 2021 to March 2022. Patients received either rivaroxaban 10 mg/day (Regimen 1) or methylprednisolone 16 mg/day plus rivaroxaban 10 mg/day (Regimen 2) for 7 days. Outcomes included progression to severe disease, adverse events, and changes in clinical and laboratory parameters. Statistical analysis employed Fisher’s exact test and Generalized Estimating Equations (GEE).

**Results:**

Among 108 patients, no progression to severe disease or deaths occurred. Regimen 2 significantly improved laboratory markers, including white blood cell counts (+0.290; p<0.001), platelet counts (+0.102; p=0.032), aspartate aminotransferase (+0.335; p=0.013), alanine aminotransferase (+0.397; p=0.006) and CD4 cells (+0.458; p<0.001), and reduced activated partial thromboplastin time (–0.083; p=0.003) compared to Regimen 1. No significant differences in clinical symptoms were observed. Adverse events were rare, with one non-serious event reported in Regimen 1.

**Conclusion:**

Combination therapy with methylprednisolone and rivaroxaban enhanced immune and coagulation parameters without increasing adverse events. These findings suggest potential benefits of early intervention in mild COVID-19, warranting further research.

## Zusammenfassung

**Zielsetzung:**

Bewertung der Wirksamkeit und Sicherheit von Methylprednisolon in Kombination mit Rivaroxaban im Vergleich zur Monotherapie mit Rivaroxaban bei Patienten mit leichter COVID-19.

**Methode:**

Die quasi-experimentelle Studie wurde in drei Krankenhäusern in Ho-Chi-Minh-Stadt, Vietnam, von September 2021 bis März 2022 durchgeführt. Die Patienten erhielten entweder Rivaroxaban 10 mg/d (Schema 1) oder Methylprednisolon 16 mg/d plus Rivaroxaban 10 mg/d (Schema 2) für 7 d. Erfasst wurden das Fortschreiten der Krankheit, unerwünschte Ereignisse und Veränderungen der klinischen und Laborparameter. Zur statistischen Analyse wurden der Fisher’s exact test und Generalisierte Schätzgleichungen (GEE) verwendet.

**Ergebnisse:**

Bei 108 Patienten kam es weder zu einem Fortschreiten der Krankheit noch zu Todesfällen. Schema 2 verbesserte signifikant die Labormarker einschließlich Anzahl der Leukozyten (+0,290; p<0,001) Thrombozyten (+0,102; p=0,032) und CD4 Zellen (+0,458; p<0,001), Anstieg der Aspart-Aminotransferase (+0,335; p=0,013) und der Alanin-Aminotransferase (+0,397; p=0,006) und senkte die aktivierte partielle Thromboplastinzeit (–0,083; p=0,003) im Vergleich zu Schema 1. Es wurden keine signifikanten Unterschiede bei den klinischen Symptomen beobachtet. Unerwünschte Ereignisse waren selten mit einem nicht schwerwiegenden Ereignis, das für Schema 1 gemeldet wurde.

**Schlussfolgerung:**

Die Kombinationstherapie mit Methylprednisolon und Rivaroxaban verbesserte die Immun- und Gerinnungsparameter, ohne die Zahl unerwünschter Ereignisse zu erhöhen. Diese Ergebnisse deuten auf einen potenziellen Nutzen der frühzeitigen Intervention bei leichter COVID-19 hin und rechtfertigen weitere Untersuchungen.

## Introduction

In July 2021, Vietnam’s Ministry of Health issued decision No. 3416/QD-BYT, updating COVID-19 treatment protocols to include low-molecular-weight heparin (LMWH) and dexamethasone for patients experiencing respiratory distress. These drugs, particularly corticosteroids and anticoagulants, have shown promising results. However, the scientific validation of these therapies remains an ongoing challenge, particularly in patients with mild cases of COVID-19.

The RECOVERY trial demonstrated that dexamethasone reduced 28-day mortality in patients requiring respiratory support, but offered no benefit to those not on supplemental oxygen. The trial utilized a dose equivalent to 32 mg of methylprednisolone daily. Subsequent studies suggested that methylprednisolone might confer superior benefits in reducing mortality compared to dexamethasone [1], [2]. Based on this evidence, low-dose methylprednisolone has been proposed as a treatment option for mild COVID-19 patients. Rivaroxaban, a highly selective direct factor XA (FXa) inhibitor, has been widely used since 2008 for venous thromboembolism (VTE) prophylaxis in post-surgical patients. It involves inhibiting free FXa, FXa within the prothrombinase complex, and clot-bound FXa without directly affecting platelet aggregation [3].

Vietnam faces significant challenges in managing COVID-19 due to shortages of medical personnel and critical care resources, particularly at tertiary hospitals. These limitations necessitate the use of effective pharmacological interventions to prevent disease progression. This study seeks to evaluate the clinical and laboratory efficacy of a combined methylprednisolone and rivaroxaban regimen in mild COVID-19 patients. This study aims to address two key objectives:

1. To compare the rate of progression to severe disease and the incidence of adverse effects in mild COVID-19 patients treated with methylprednisolone and rivaroxaban versus rivaroxaban monotherapy.
2. To evaluate changes in clinical symptoms and laboratory parameters between the two treatment groups.

## Method

### Study design

The study was conducted as quasi-experimental, open-label study at three hospitals: Hoan My Thu Duc Hospital, Hoan My Van Phuc 1 Hospital, and Hoan My Van Phuc 2 Hospital, all located in Ho Chi Minh City, Vietnam. The study was conducted in the period from September 2021 to March 2022, concluding when the required sample size was reached.

The sample size was calculated using a formula for comparing two proportions. Based on the expected differences in the progression rate to severe disease between treatment groups, a total sample size of 103 participants was estimated. The specific formula used for the calculation was:


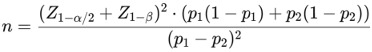


Where 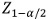 represents the critical value for a two-sided test (α=0.05), 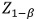 accounts for 80% power, and *p1* and *p2* are the expected proportions in each group.

Participants were assigned to one of two treatment regimens by doctors following national guidelines:

- Regimen 1 (Control Group): Rivaroxaban (Xarelto) 10 mg once daily for 7 days.
- Regimen 2 (Experimental Group): Methylprednisolone (Medrol) 16 mg once daily for 7 days, combined with Rivaroxaban 10 mg once daily for 7 days.

Supportive medications such as paracetamol, eucalyptus cough syrup, and vitamins permitted by the Ministry of Health were allowed, while nonsteroidal anti-inflammatory drugs (NSAIDs) were avoided to minimize confounding effects.

### Participants

Eligible participants were adult patients (aged 18–65 years) diagnosed with mild COVID-19 according to the Ministry of Health guidelines as of September 2021. Inclusion criteria included:

- Positive SARS-CoV-2 RT-PCR test,
- Meeting hospitalization criteria with SpO_2_ ≥96% and respiratory rate <20 breaths/minute.

Exclusion criteria included:

- Receipt of antiviral, anti-inflammatory, or anticoagulant treatment before hospital admission,
- moderate, severe, or critical COVID-19 cases as per Ministry of Health guidelines,
- history of allergies to methylprednisolone, hydrocortisone, dexamethasone, or excipients.
- epilepsy or bleeding disorders (e.g., bleeding ulcers within the past 3 months, subcutaneous or mucosal bleeding),
- renal failure (glomerular filtration rat e<30 mL/minute) or liver failure,
- diagnosed immune disorders or ongoing corticosteroid treatment,
- radiotherapy or pregnancy,
- refusal to provide informed consent.

### Variables and data collection

Data recorded at admission and during hospitalization collected included patient demographics, medical history, comorbidities, medication use, symptom onset, admission symptoms, SpO_2_, and laboratory test results (WBC, PLT, AST, ALT, CD4, APTT).

### Statistical analysis

The data were analyzed using Stata 17.0, with results presented according to statistical standards for biomedical research. Continuous variables, such as age, body mass index (BMI), symptom onset-to-treatment interval, and laboratory parameters, were summarized as means and standard deviations (SD), while categorical variables, such as gender, vaccination status, and adverse events, were reported as absolute frequencies and percentages. Statistical significance was defined as p<0.05, with all tests two-tailed. To compare baseline characteristics between the two treatment groups, independent sample t-tests were used for continuous variables, and chi-square tests were applied for categorical variables, as shown in Table 1 (Tab. 1). Fisher’s exact test was substituted when the assumptions of the chi-square test were not met.

For the first study objective, adverse event rates were analyzed using chi-square tests to determine differences between the regimens (Table 2 (Tab. 2)). For the second objective, changes in clinical symptoms over time were evaluated using Generalized Estimating Equations (GEE) models, as detailed in Table 3 (Tab. 3). Univariate GEE models were used to assess the time-dependent effect of treatment regimens, while multivariate GEE models adjusted for potential confounders, including age, sex, vaccination status, baseline symptoms, and days from symptom onset to treatment. Laboratory parameters, including immune and coagulation indices, were analyzed to compare the two regimens (Table 4 (Tab. 4)). Changes in white blood cell (WBC) count, platelet (PLT) count, aspartate aminotransferase (AST), alanine aminotransferase (ALT), activated partial thromboplastin time (APTT), and CD4 counts were assessed using GEE models, which account for repeated measurements within individuals and adjust for relevant covariates. The GEE models used an exchangeable working correlation structure, assuming equal correlation between repeated measures within each subject. Regression coefficients (β) from these models represented the estimated change in the outcome variable associated with the treatment regimen, adjusted for other variables. For example: A positive beta coefficient (β>0) indicates an increase in the outcome variable (e.g., WBC count) for the experimental group (Regimen 2) compared to the control group (Regimen 1). A negative beta coefficient (β<0) indicates a decrease in the outcome variable (e.g., APTT) for the experimental group compared to the control group (Regimen 1). The magnitude of β reflects the strength of the effect. For instance, β=+0.290 for WBC count (p<0.001) in the experimental group signifies an average increase of 0.290 G/L compared to the control group, after controlling for confounding factors. Confidence intervals (CI) for β coefficients were calculated to assess precision, and p-values indicated whether changes were statistically significant. For instance, an APTT beta coefficient of –0.083 (95% CI: –0.137 to –0.028, p=0.003) for the experimental group indicates a statistically significant reduction in APTT compared to the control group.

## Results

108 participants were surveyed in August 2021: 49 participants received regimen 1 and the remaining 59 others were assigned regimen 2. Table 1 (Tab. 1) summarizes the demographic and clinical characteristics of the participants. The mean age was 33.2 years (SD 11.43), and the majority of participants were male (60.9%). The most common admission symptoms were dry cough (43.9%), muscle pain (21.4%), and fever >38.5°C (20.4%). Most participants had received one dose of a COVID-19 vaccine (46.3%).

Table 2 (Tab. 2) addresses the first study objective, focusing on the comparison of adverse events between the two treatment groups. The results demonstrate no statistically significant difference in the occurrence of adverse events between Regimen 1 (Rivaroxaban monotherapy) and Regimen 2 (Methylprednisolone plus Rivaroxaban). Specifically, only one participant in the monotherapy group experienced a non-serious adverse event related to Rivaroxaban, while no adverse events were reported in the combination therapy group. The p-value derived from Fisher’s exact test (p=0.270) indicates that the observed difference in adverse event rates between the groups is not statistically significant.

Changes in clinical symptoms between the two groups, analyzed using the Generalized Estimating Equations (GEE) model, are presented in Table 3 (Tab. 3). No significant differences were observed between the groups for body temperature, respiratory rate, SpO_2_, heart rate, or blood pressure after adjusting for confounders such as age, sex, vaccination status, and baseline symptoms.

On the 7^th^ day, patients in Regimen 2 had significantly higher white blood cell (WBC) counts (10.49±3.18) than did those in Regimen 1 (7.1±2.12), indicating an enhanced immune response. Similarly, platelet (PLT) levels were elevated in Regimen 2 (279.17±64.2 vs. 257.12±66.8), suggesting better coagulation support. Liver enzyme levels were also higher in Regimen 2, with AST at 30.31±20.41 and ALT at 32.66±23.62 compared to 28.74±17.95 and 24.9±17.59 in Regimen 1, respectively. This mild increase may reflect hepatic stress or metabolic activation associated with the combination therapy. Immune function, as measured by CD4 counts, showed substantial improvement in Regimen 2 (1270.36±470.33 vs. 1016.07±334.08 in Regimen 1), indicating better preservation or recovery of immune competence. Furthermore, activated partial thromboplastin time (APTT) was significantly lower in Regimen 2 (35.37±6.87 vs. 41.36±9.73), reflecting improved coagulation balance.

Table 5 (Tab. 5) shows the changes in blood biochemical parameters over time from admission to 28 days post-treatment. In the univariate GEE model, patients receiving regimen 2 had lower D-dimer levels and higher CD8 levels compared to those receiving regimen 1, but these differences were not statistically significant in the multivariate model. Improvements in laboratory parameters were observed across both groups, with significant differences favoring the combination therapy group (Regimen 2) (Table 4 (Tab. 4)). Patients in Regimen 2 had higher WBC levels (+0.290; 95% CI 0.148 to 0.432; p<0.001), PLT levels (+0.102; 95% CI 0.008 to 0.195; p=0.032), AST levels (+0.335; 95% CI 0.070 to 0.599; p=0.013), ALT levels (+0.397; 95% CI 0.115 to 0.680; p=0.006), and CD4 counts (+0.458; 95% CI 0.259 to 0.656; p<0.001). APTT levels were lower in the combination therapy group (–0.083; 95% CI –0.137 to –0.028; p=0.003). Patients receiving regimen 2 (combined regimen) had lower APTT levels by –0.083 (–0.137; –0.028) with p-value=0.003 compared to patients receiving regimen 1 (monotherapy).

## Discussion

While no patients in either group progressed to severe disease or experienced significant adverse events, the combination therapy group demonstrated notable improvements in several laboratory parameters. Specifically, patients receiving methylprednisolone and rivaroxaban had significantly higher WBC, PLT, CD4 levels, AST and ALT, alongside reduced APTT compared to the monotherapy group. However, there were no statistically significant differences in clinical symptom improvement or in the multivariate analysis of D-dimer and CD8 levels, suggesting that the observed laboratory changes may not fully translate into short-term clinical benefits. Further investigation is warranted to assess the long-term implications of these findings.

Our results align with the RECOVERY trial, which demonstrated the efficacy of dexamethasone in reducing 28-day mortality among COVID-19 patients requiring oxygen or mechanical ventilation, though benefits were not observed in those without respiratory support [4]. Similarly, Ranjbar et al. [5] found that methylprednisolone outperformed dexamethasone in reducing inflammation and mortality in hospitalized COVID-19 patients. 

The anticoagulant properties of rivaroxaban offer an additional therapeutic advantage in managing COVID-19. COVID-19-associated coagulopathy, characterized by elevated D-dimer levels and an increased risk of thromboembolic events, remains a critical complication. Interestingly, while several studies have demonstrated that anticoagulants like rivaroxaban reduce the risk of thrombosis and mortality in severe cases, others, such as the COVID-PREVENT trial, reported no significant clinical improvements or reductions in thrombotic risk associated with rivaroxaban [6]. We provide evidence of rivaroxaban’s short-term thrombosis-reducing effects when combined with methylprednisolone, as reflected by improved coagulation parameters, including lower APTT, in the combination therapy group. However, previous studies that refuted rivaroxaban’s thrombotic benefits may have been affected by significant selection bias due to high loss-to-follow-up rates [7]. In contrast, our study experienced minimal loss to follow-up during the observation period, thereby providing more robust evidence. Thus, the observed reduction in thrombotic risk with rivaroxaban, as supported by laboratory parameter improvements rather than direct clinical outcomes, appears biologically plausible. Nonetheless, further studies are warranted to confirm these findings and to explore the long-term clinical implications of rivaroxaban in COVID-19 management.

Corticosteroids play a critical role in managing inflammation by inhibiting cytokine production, thereby exerting potent anti-inflammatory effects. Their use extends beyond intensive care unit (ICU) patients; studies have demonstrated that administering steroids to non-ICU COVID-19 patients is associated with reduced rates of ICU admission, intubation, and mortality [8]. Among corticosteroids, methylprednisolone and dexamethasone have been widely utilized, with evidence showing improved outcomes for hospitalized COVID-19 patients [5]. Previous research has underscored the efficacy of corticosteroids in reducing mortality in COVID-19 patients requiring oxygen therapy or mechanical ventilation within 28 days, with a pooled odds ratio (OR) of 0.66 (95% CI: 0.53–0.82; p<0.001). Despite this, mortality remains significant at 32.7% in these populations [8]. An observational study on non-severe COVID-19 patients found that the combination of corticosteroids (equivalent to 1.25 mg/kg/24 hours of prednisolone) and furosemide (80 mg/day) administered within four days of symptom onset reduced the need for mechanical ventilation and overall mortality (OR: 0.35; 95% CI: 0.11–1.01; p=0.04) [9]. Similarly, a study by Canaan et al. [10] investigated 121 mechanically ventilated ICU patients with COVID-19. Although the methylprednisolone group had lower in-hospital mortality rates (p=0.381) and shorter hospital stays (p=0.307) compared to the dexamethasone group, these differences were not statistically significant. Survival analysis and Cox proportional regression models also indicated lower, albeit not significant, in-hospital mortality in the methylprednisolone group (HR: 0.64; 95% CI: 0.35–3.17).

In our study, conducted over a 30-day follow-up period, no deaths or progression to severe disease were observed in either treatment regimen. Laboratory findings revealed that patients receiving Regimen 2 (methylprednisolone plus rivaroxaban) exhibited superior immune and coagulation profiles compared to Regimen 1 (rivaroxaban monotherapy). Univariate analysis showed lower D-dimer levels and higher CD8 counts in the combination group, although these differences were not statistically significant in the multivariate model. Regimen 2 significantly improved WBC levels (+0.290; 95% CI: 0.148–0.432; p<0.001), platelet (PLT) counts (+0.102; 95% CI: 0.008–0.195; p=0.032), AST) levels (+0.335; 95% CI: 0.070–0.599; p=0.013), ALT levels (+0.397; 95% CI: 0.115–0.680; p=0.006), and CD4 counts (+0.458; 95% CI: 0.259–0.656; p<0.001). Additionally, APTT was significantly lower in Regimen 2 (-0.083; 95% CI: -0.137 to -0.028; p=0.003). Across both regimens, laboratory parameters improved regardless of treatment group. Among the study participants, the majority were male (60.9%), and nearly half had received one dose of the COVID-19 vaccine (46.30%). The most frequently reported admission symptoms included cough (43.88%), muscle pain (21.43%), and fever >38.5°C (20.41%). No participants progressed to severe disease, and no deaths occurred after 28 days of follow-up.

### Limitations

Despite these promising results, the study has limitations. The quasi-experimental design lacks the rigor of randomized controlled trials (RCTs), and the short follow-up period limits the ability to assess long-term outcomes such as post-COVID sequelae. Moreover, the study excluded high-risk groups, such as patients with comorbidities or severe disease, which may restrict the generalizability of the findings. Future RCTs with larger, more diverse populations and extended follow-up periods are needed to validate these results and explore the broader clinical implications of this combination therapy.

### Strengths

The study has several strengths, including its focus on a practical and scalable treatment approach for mild COVID-19, addressing a critical gap in preventing disease progression, particularly in resource-limited settings. It compares combination therapy (methylprednisolone and rivaroxaban) with monotherapy, offering valuable insights into managing the inflammatory and thrombotic aspects of COVID-19. The detailed analysis of laboratory parameters provides mechanistic understanding, while the well-defined patient cohort minimizes variability, enhancing internal validity. Conducted in real-world clinical settings, the findings are highly applicable to routine healthcare. 

### Clinical Applications

The study highlights the potential of combining methylprednisolone and rivaroxaban as an effective treatment for mild COVID-19. The regimen addresses key inflammatory and thrombotic components, with improved laboratory parameters such as higher WBC, PLT, and CD4 levels, and reduced APTT, indicating enhanced immune modulation and anticoagulation. Importantly, no patients progressed to severe disease, emphasizing the role of early intervention in preventing complications. The therapy offers a practical, scalable option for resource-limited settings, reducing the burden on healthcare systems. These findings provide a basis for revising treatment protocols and conducting further research to validate its broader clinical use.

## Conclusion

The study investigated methylprednisolone combined with rivaroxaban versus rivaroxaban monotherapy in treating mild COVID-19. None of the 108 participants progressed to severe disease post-treatment. While adverse effects were rare, the combined regimen (Regimen 2) showed significantly higher WBC, platelets, AST, ALT, and CD4 levels, alongside with lower APTT levels compared to monotherapy (Regimen 1). There were no significant differences in clinical symptoms between groups. Laboratory parameters improved regardless of the regimen, with no significant differences in D-dimer and CD8 levels in multivariate analysis. Further research is needed to validate these findings.

## Notes

### Author’s ORCID

- Nguyen Thien M: 0009-0003-1090-6228

### Ethical approval 

The study was approved by the Ethics Committee for Biomedical Research of Hoan My Medical Corporation in August 18^th^, 2021, No. 23/2021/GCN-HDDD-HM. All patients provided written informed consent after being fully briefed on the study objectives, medications, and potential risks. Participants were assured that withdrawal from the study would not affect their standard care. Patient confidentiality was strictly maintained, with all data used solely for research purposes. Clinical examinations and laboratory tests were conducted per Ministry of Health guidelines.

### Funding

This work was supported by Hoan My Medical Group, Vietnam, grant number 11/2021/HM-GFR.

### Competing interests

The authors declare that they have no competing interests.

## Figures and Tables

**Table 1:**
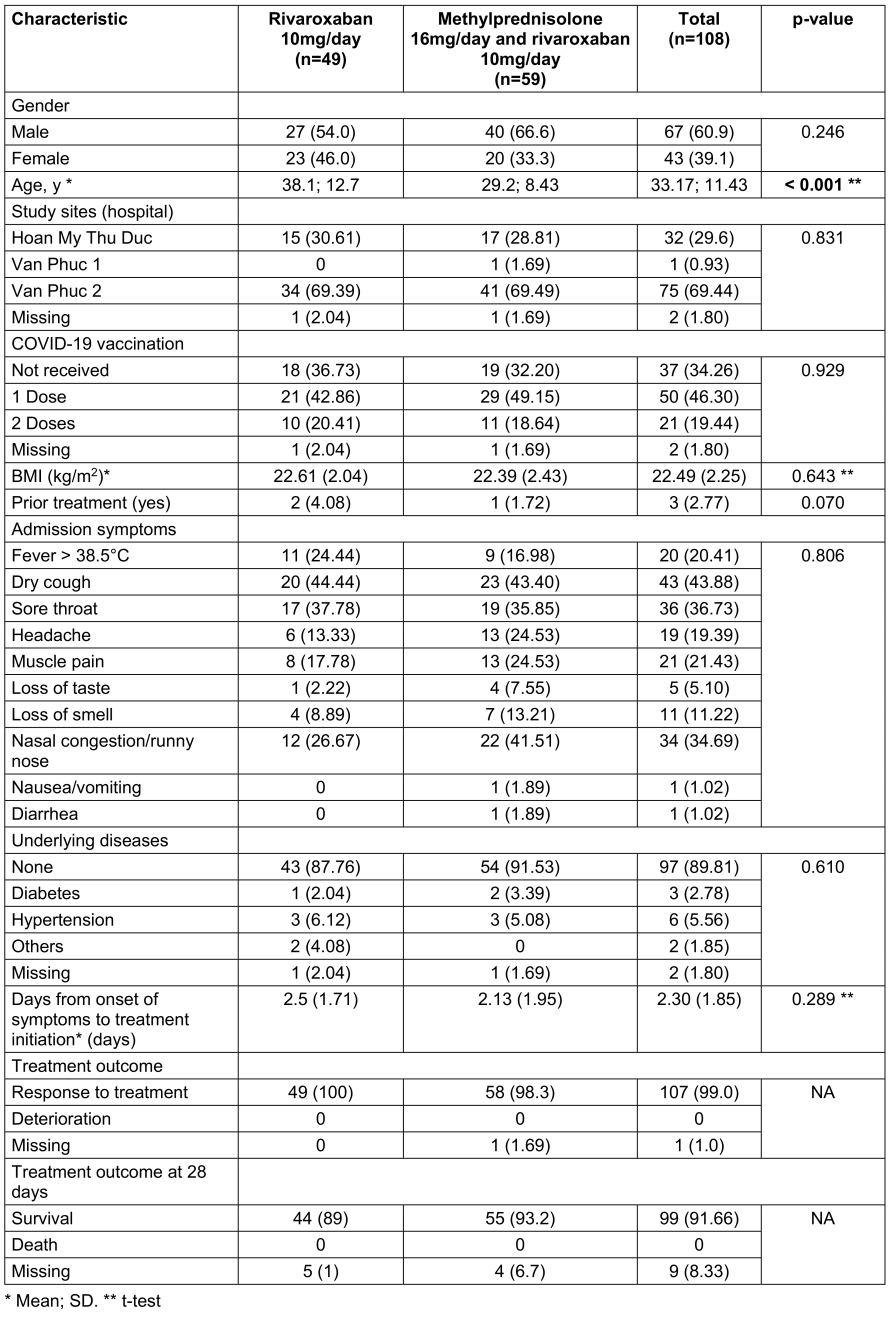
Demographic characteristics, admission symptoms, underlying diseases, and treatment outcomes of participants

**Table 2:**
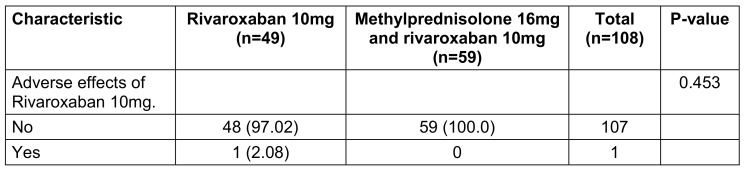
Adverse effects when using medication according to the study protocol

**Table 3:**
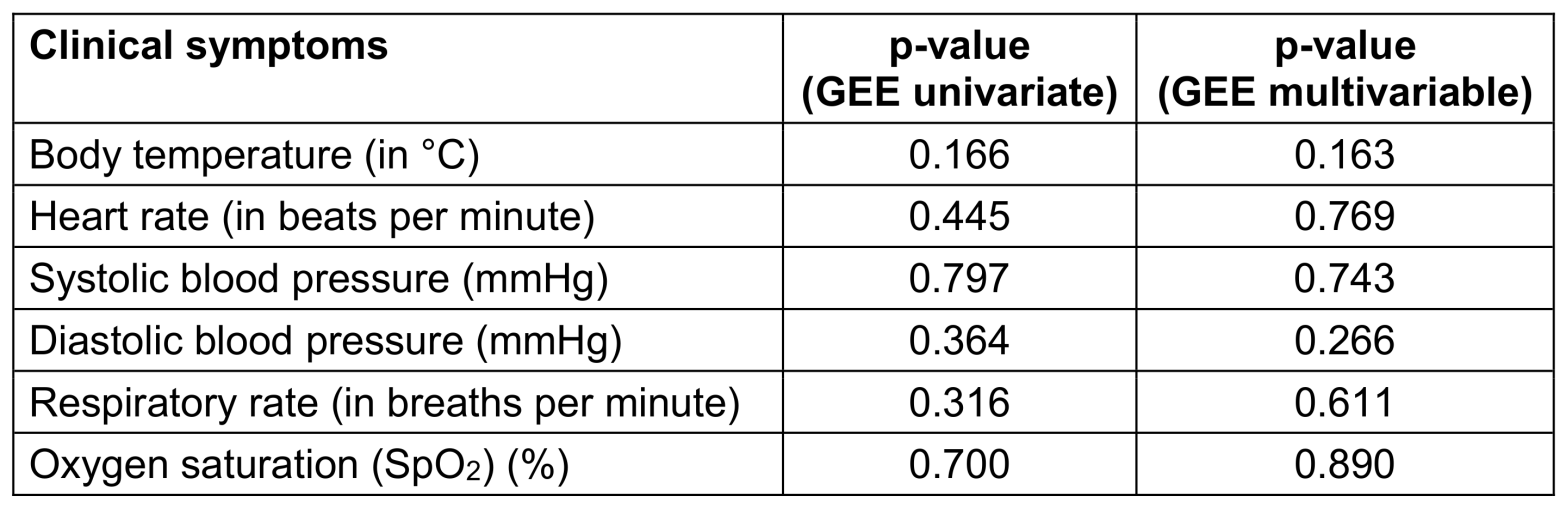
Comparison of changes in clinical symptoms between the two regimens (n=108)

**Table 4:**
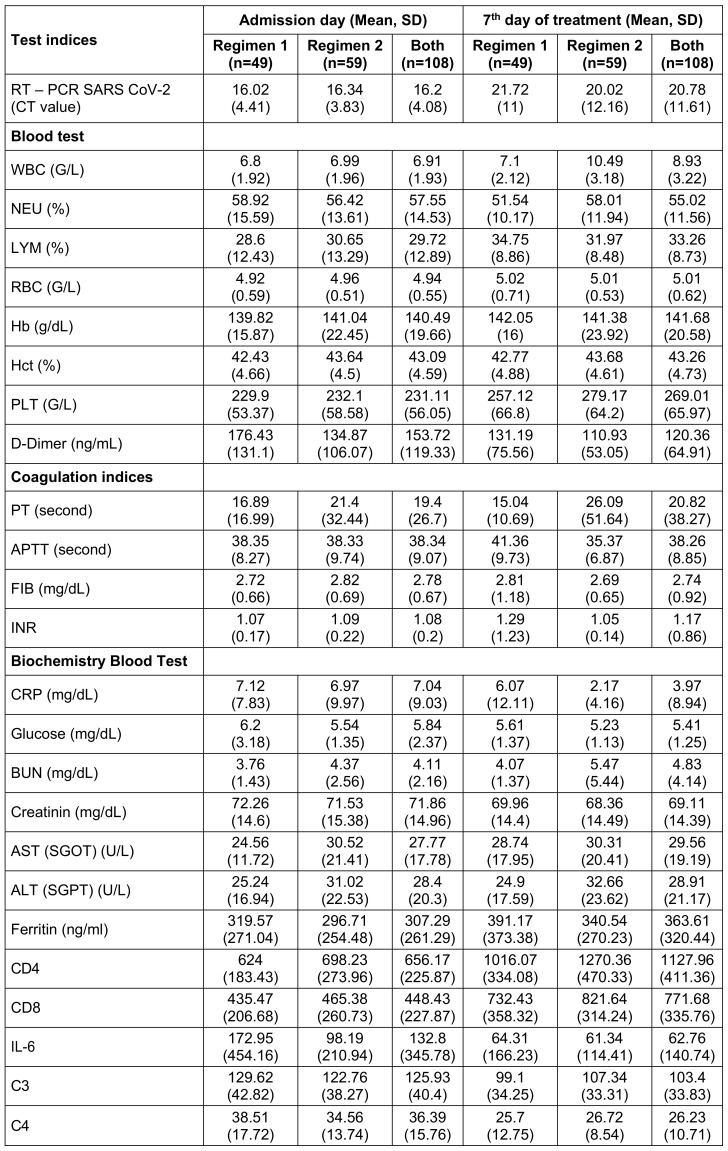
The characteristics of the laboratory indices of the patients in the two regimens on the admission day and on the 7^th^ treatment day (n=108).

**Table 5:**
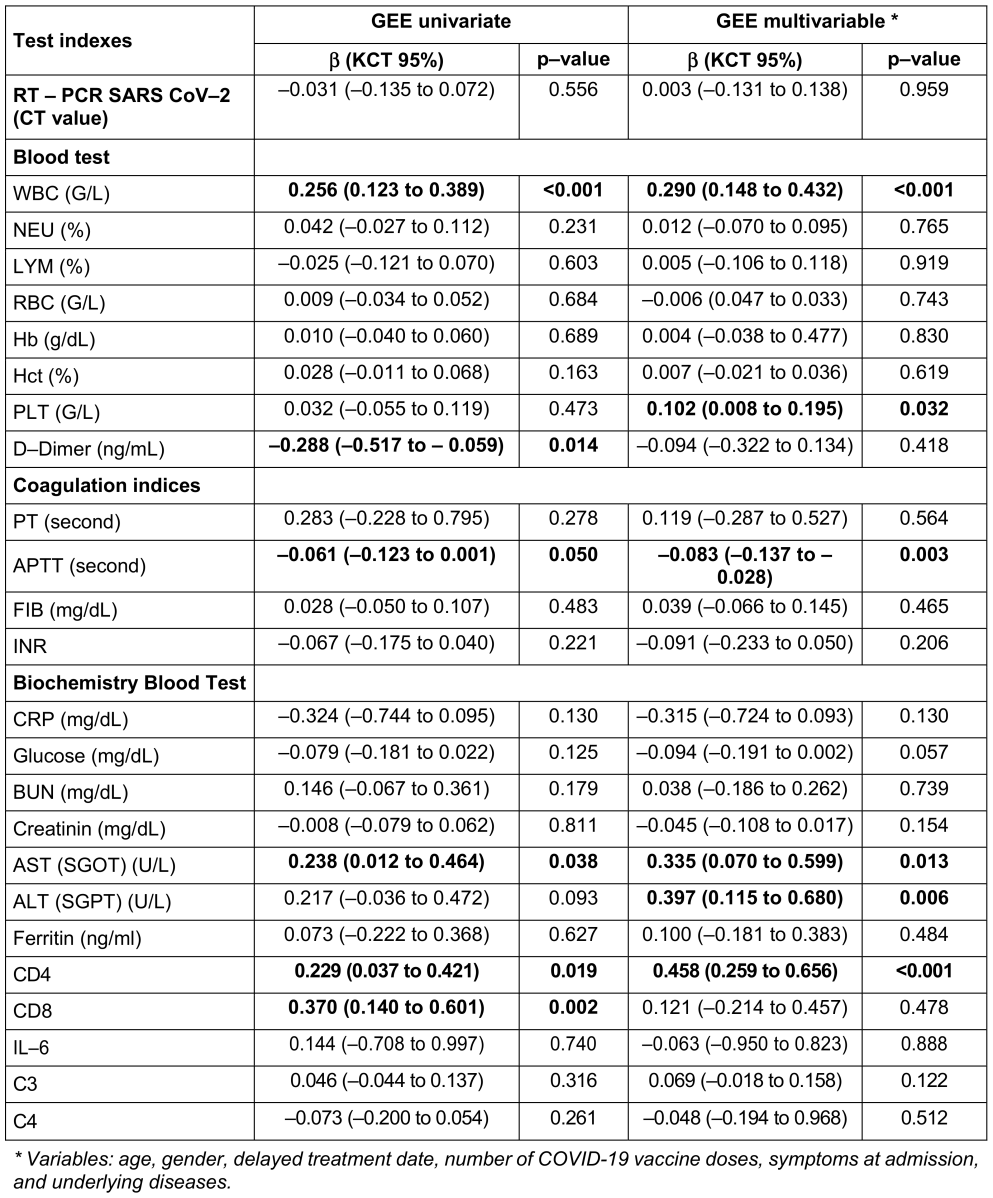
Comparison of changes of paraclinical indicators in study participants, univariate and multivariate GEE models up to 28 days (n=108)
